# Supplementary material for: Development of New Iso-Cytoplasmic Rice-Restorer Lines and New Rice Hybrids with Superior Grain Yield and Grain Quality Characteristics by Utilizing Restorers’ Fertility Genes
Source: Genes (Basel). 2022 May 1;13(5):808. doi: 10.3390/genes13050808 (PMC9140396; doi:10.3390/genes13050808)
Supplement: Supplementary file 1 [file genes-13-00808-s001.zip › genes-1689570-supplementary.pdf]

Table S1. Mean performance for the grain yield, floral traits, and its contributing traits of the studied genotypes

Traits / Genotypes	PF (%)	SF (%)	DH	PH (cm)	P/P	PL (cm)	Sp/P	FG/P	PW (g)	1000 GW (g)	GY/P (g)	AL	AB	FL
G46A*	93.98	90.86	80.03	86.73	10.77	23.18	177.53	166.8	3.9	25.95	37.89	1.8	0.35	6.37
IR69625A*	93.99	90.93	99.7	102.33	14.3	21.95	135	126.63	3.79	25.21	39.07	1.83	0.38	6.17
NRL79	94.81	91.09	102	111.9	14.03	25.63	281.8	257.4	6.25	24.83	54.5	2.58	0.48	7.1
NRL80	94.65	91.59	104	119.1	15.17	25.85	238.5	220.9	6.39	24.44	51.6	2	0.46	6.94
NRL81	98.09	95.44	105	119.4	19.5	21.39	170.45	166.1	4.83	25.43	66.96	1.87	0.47	6.77
NRL82	94.23	91.13	106.7	136.3	20.83	27.74	204.75	191.3	5.61	27.33	68.5	2.92	0.56	7.6
Giza 178	93.73	90.48	101.9	103.67	13.43	22.85	165.57	156.84	3.9	20.83	34.18	1.76	0.4	3.97
G46A × NRL79	94	90.83	101.02	109.33	16.43	24.47	223	205.21	5.67	25.18	48.26	2.34	0.43	6.95
IR69A × NRL79	93.99	90.16	102.4	103	29	24.47	198.4	179.23	4.68	24	57	2.41	0.48	6.8
G46A × NRL80	91.7	88.25	102	110.17	28.13	25.28	271.17	231.53	5.1	25.43	64.63	2.01	0.43	6.92
IR69A × NRL80	98.22	95.08	103.89	106.17	13.4	22.06	138.6	135.22	3.72	25.6	44.07	2.1	0.46	6.77
G46A × NRL81	98.02	94.93	102.01	124.17	29.83	25.6	206.2	199.39	6.18	28.18	87.14	1.86	0.42	6.97
IR69A × NRL81	96.82	91.31	104.01	102.67	13.73	23.46	165.13	153.28	4.42	26.93	47.76	1.9	0.45	6.83
G46A × NRL82	92.56	88.46	101.01	119	27.13	24.45	192.83	164.19	4.15	28.78	41.55	2.52	0.49	7.51
IR69A × NRL82	91.57	87.35	105.7	103.33	21.5	22.53	167	140.23	3.68	26.59	45	2.56	0.53	7.39
G46A × Giza 178	91.37	88.87	102.7	123.37	15.37	24.07	309.33	287.79	4.88	25.27	43.84	1.91	0.41	5.1
IR69A × Giza 178 ‡	93.19	89.38	101.01	106.3	18.73	22.73	158.63	154.17	4.13	23.83	38.72	1.87	0.38	4.92
L.S.D. 5%	1.93	2.13	0.86	2.6	2.22	0.8	18.18	20.21	0.71	0.84	4.83	0.42	0.05	0.58
L.S.D. 1%	2.59	2.86	1.15	3.49	2.99	1.07	24.44	27.16	0.95	1.13	6.49	0.56	0.07	0.78
CV	1.42	1.63	0.59	1.62	8.17	2.31	6.3	7.6	10.26	2.29	6.55	13.77	7.94	6.13

*: The traits were recorded on maintainer lines which were related to CMS lines

‡: Hybrid check

PF : Pollen fertility percentage, SF: Spikelet fertility percentage, HD: Days to 50% heading (day), PH: Plant height (cm), PL: Panicle length (cm), P/P: Number of panicles per plant, Sp/P: Number of spikelets per panicle, FG/P: Number of filled grains per panicle, PW: Panicle weight (g), 1000-GW: 1000-grain weight (g), GY/P: Grain yield per plant (g), AL: Anther length (mm), AB: Anther breadth (mm), and FL: Filament length (mm),

Table S2. Mean performance for the grain quality traits of the studied genotypes

Traits / Genotypes	GL	GW	GS	KL	KW	KS	GE	H (%)	M (%)	HR (%)
G46A*	8.4	3.08	2.72	5.03	2.67	1.89	84.18	78.12	67.23	64.95
IR69625A*	8.17	2.63	3.1	5.6	2.23	2.51	67.61	79.14	69.24	60.77
NRL79	6.95	3.05	2.28	5.13	2.75	1.87	97.17	79.73	71.28	64.01
NRL80	7.7	3.1	2.49	5	2.52	1.99	98.7	81.77	72.11	67.75
NRL81	7.8	3	2.6	5.3	2.63	2.01	124.32	82.8	73.82	68.58
NRL82	7.87	3.22	2.45	5.07	2.85	1.78	119.41	81.35	71.6	67.36
Giza 178	7.32	2.72	2.69	4.98	2.38	2.09	80.16	81.68	71.74	68.99
G46A × NRL79	7.63	3.2	2.39	5.17	2.83	1.83	58.28	78.8	69.2	58.33
IR69A × NRL79	7.55	2.9	2.6	5.38	2.56	2.1	76.16	78.47	68.27	59.27
G46A × NRL80	7.47	3.18	2.35	5.2	2.63	1.98	73.14	81.53	72.6	69.73
IR69A × NRL80	7.83	2.98	2.63	5.37	2.48	2.17	57.64	78.13	66	60
G46A × NRL81	7.67	3.38	2.27	5.42	2.85	1.9	52.98	80.07	73.33	69
IR69A × NRL81	7.95	3.1	2.57	5.43	2.65	2.05	71.84	80.33	73.67	57
G46A × NRL82	7.73	3.2	2.42	5.43	2.68	2.02	50.48	80.87	73.73	73
IR69A × NRL82	7.75	3.07	2.53	5.47	2.54	2.15	59.39	79.47	69.93	53.93
G46A × Giza 178	7.62	3.15	2.42	5.41	2.69	2.01	90.8	80.01	69.32	63.3
IR69A × Giza 178 ‡	7.81	2.77	2.82	5.55	2.48	2.24	69.09	79.42	69.4	58.44
L.S.D. 5%	0.24	0.1	0.13	0.15	0.1	0.1	3.2	1.33	1.44	2.66
L.S.D. 1%	0.33	0.14	0.18	0.2	0.14	0.14	4.3	1.79	1.94	3.58
CV	2.19	2.38	3.57	1.93	2.69	3.56	2.84	1.15	1.42	2.9

*: The traits were recorded on maintainer lines which were related to CMS lines

‡: hybrid check

GL: Grain length, GW: Grain width, GS: Grain shape, KL: kernel length, KW: Kernel width, KS: Kernel shape, GE: Grain elongation, H: Hulling percentage, M: Milling percentage and HR: Head rice recovery percentage.

Table S3. Analysis of variance and combining ability for the grain yield, floral traits, and its contributing traits for genotypes studied.

Source of variance (SOV)	Replications	Genotypes	Parents	P. vs. Crosses	Crosses	Lines	Tester	Line × Tester	Error
d.f	2	16	6	1	9	1	4	4	32
Pollen fertility (%)	0.04ns	14.60**	6.82**	5.05ns	20.85**	11.27ns	28.85**	15.24**	1.79
Spikelet fertility (%)	3.71ns	16.46**	8.72**	17.37**	21.51**	1.13ns	25.25*	22.87**	2.19
Days to heading	0.004ns	101.83**	246.10**	87.99**	7.19**	20.55**	3.30*	7.75**	0.35
Plant height (cm)	0.15ns	398.68**	746.99**	4.41ns	210.27**	1250.66**	77.46**	82.99**	3.25
No. of Panicles/plant	3.63ns	115.46**	37.40**	429.03**	132.67**	126.49**	44.53**	222.36**	2.39
Panicle length (cm)	0.15ns	8.57**	16.49**	0.37ns	4.19**	22.26**	1.78ns	2.09**	0.31
No. of spikelets/ panicle	65.4ns	7458.7**	7442.5**	464.9ns	8246.5**	41835.7**	2488.1*	5607.6**	159.3
No. of filled grains/panicle	42.3ns	5768.2**	5723.3**	113.3ns	6426.4**	32314.6**	2746.8*	3634.0**	194.4
Panicle weight (g)	0.1ns	2.6**	3.9**	1.0*	2.0**	8.6**	2.0*	0.4ns	0.2
1000 grain weight (g)	0.2ns	10.0**	12.1**	15.5**	8.0**	10.4**	14.4**	1.1*	0.3
Grain Yield/plant (g)	1.6ns	579.6**	584.1**	24.6ns	638.3**	838.8**	653.2**	573.3**	11.2
Anther length	0.158 ns	0.51**	0.98**	0.19 ns	0.24*	0.01 ns	0.53 ns	0.004 ns	0.083
Anther breadth	0.0003 ns	0.01**	0.02**	0.0004ns	0.01**	0.004 ns	0.01*	0.001 ns	0.001
Filament length	0.187 ns	2.91**	4.17**	0.50 ns	2.34**	0.16 ns	5.21**	0.001 ns	0.16

**: Highly significant at 1%   *: Significant at 5%   ns: Non-significant.

Table S4. Analysis of variance and combining ability for the grain quality traits of genotypes studied.

Source of variance (SOV)	d.f	GL	GW	GS	KL	KW	KS	GE	H (%)	M (%)	HR (%)
Replications	2	0.04ns	0.01ns	0.003ns	0.03ns	0.002ns	0.01ns	11.88ns	0.42ns	0.03ns	2.65ns
Genotypes	34	0.31**	0.11**	0.13**	0.12**	0.08**	0.09**	1433.74**	5.79**	17.07**	88.68**
Parents	12	0.72**	0.14**	0.21**	0.15**	0.14**	0.17**	1277.47**	8.48**	13.73**	26.44**
P. vs. Crosses	1	0.02ns	0.18**	0.18**	0.62**	0.05**	0.01ns	11085.39**	11.04**	2.58ns	183.86**
Crosses	21	0.06ns	0.09**	0.08**	0.04**	0.05**	0.05**	465.52**	3.42**	20.91**	119.60**
Lines	1	0.18ns	0.50**	0.51**	0.10*	0.28**	0.29**	21.29ns	8.93*	35.69**	600.05**
Tester	10	0.04ns	0.07*	0.03ns	0.06ns	0.04*	0.03ns	497.49**	2.43ns	24.93**	34.01*
Line × Tester	10	0.05ns	0.01ns	0.02ns	0.01ns	0.004ns	0.01ns	544.61**	3.03*	13.20**	85.06**
Error	68	0.03	0.01	0.01	0.01	0.005	0.01	4.94	0.85	1.01	3.42

GL: Grain length, GW: Grain width, GS: Grain shape, KL: Kernel length, KW: Kernel width, KS: Kernel shape, GE: Grain elongation, H: Hulling percentage, M: Milling percentage and HR: Head rice recovery percentage.

Table S5. Estimates of variability parameters for the grain yield, floral traits, grain quality, and their contributing traits in promising restorer lines of rice.

Crosses / Traits	GCV (%)	PCV (%)	GA	Heritability (%)	GA%
Days to heading	1.94	2	4.04	94.12	3.89
Plant height	10.18	10.24	24.63	98.84	20.86
No. of Panicles/plant	19.31	21.92	5.81	77.61	35.04
Panicle length	10.18	10.48	5.04	94.48	20.39

Pollen fertility %	1.73	1.96	2.98	77.59	3.14
No. of spikelets/panicle	22.18	24.48	87.86	82.08	41.4
Spikelet fertility (%)	2.1	2.29	3.64	83.8	3.96
No. of filled Grains/panicle	19.49	23.16	67.06	70.82	33.78
Panicle weight	18.98	19.74	2.03	92.41	37.58
1000- grain weight	9.56	9.78	4.73	95.53	19.24
Grain yield (gm/P)	24.9	25.68	27.44	94.05	49.75
Anther length (mm)	29.93	36.02	1.08	69.05	51.23
Anther breadth (mm)	11.54	13.11	0.1	77.46	20.92
Filament Length (mm)	21.36	23.74	2.56	80.91	39.57
Hulling (%)	1.27	1.54	1.75	67.71	2.15
Milling (%)	1.31	1.52	1.69	74.73	2.34
Head rice (%)	2.78	3.2	3.34	75.18	4.96
Grain length	5.03	5.34	0.73	88.55	9.74
Grain width	5.86	6.73	0.32	76.01	10.53
Grain types	6.1	6.5	0.3	88.09	11.79
Grain (kernel) length	2.33	2.78	0.21	70.42	4.03
Grain (kernel) width	6.68	7.69	0.31	75.56	11.96
Grain elongation	17.2	17.48	36.26	96.85	34.88
Grain (kernel) types	6.14	6.78	0.22	81.87	11.44

(PCV%): Phenotypic Coefficient of Variation, (GCV%): Genotypic Coefficient of Variation GA: Genetic Advance and GA%: Genetic Advance

Table S6. Estimates of the percentage of heterosis over better-parent (BP) for grain yield, its contributing and floral traits of studied hybrids.

Traits / crosses	PF (%)	SF (%)	DH	PH (cm)	P/P	PL (cm)	Sp/P	FG/P	PW (g)	1000 GW (g)	GY/P (g)	AL	AB	FL
G46A × NRL79	-0.9ns	-0.3ns	26.2**	26.1**	17.1ns	-4.5*	-20.9**	-20.3**	-9.3ns	-3.0ns	-11.4*	-9.4ns	-10.4ns	-2.1ns
IR69A × NRL79	-0.9ns	-1.0ns	2.7**	0.7ns	102.8**	-4.5*	-29.6**	-30.4**	-25.1**	-4.8*	4.6ns	-6.7ns	0.0ns	-4.2ns
G46A × NRL80	-3.1*	-3.6**	27.4**	27.0**	85.5**	-2.2ns	13.7**	4.8ns	-20.1**	-2.0ns	25.3**	0.3ns	-6.5ns	-0.3ns
IR69A × NRL80	3.8**	3.8**	4.2**	3.7*	-11.6ns	-14.7**	-41.9**	-38.8**	-41.8**	1.5ns	-14.6**	4.8ns	0.0ns	-2.4ns
G46A × NRL81	-0.1ns	-0.5ns	27.5**	43.2**	53.0**	10.4**	16.1**	19.5**	28.0**	8.6**	30.1**	-0.4ns	-10.6ns	3.0ns
IR69A × NRL81	-1.3ns	-4.3**	4.3**	0.3ns	-29.6**	6.9**	-3.1ns	-7.7ns	-8.4ns	5.9**	-28.7**	1.8ns	-4.3ns	0.9ns
G46A × NRL82	-1.8ns	-2.9*	26.2**	37.2**	30.2**	-11.8**	-5.8ns	-14.2*	-26.0**	5.3**	-39.3**	-13.8ns	-12.5*	-1.2ns
IR69A × NRL82	-2.8*	-4.2**	6.0**	1.0ns	3.2ns	-18.8**	-18.4**	-26.7**	-34.4**	-2.7ns	-34.3**	-12.4ns	-5.4ns	-2.8ns
G46A × Giza 178	-2.8*	-2.2ns	28.3**	42.2**	14.4ns	3.8ns	74.2**	72.5**	25.1*	-2.6ns	15.7*	6.3ns	2.5ns	-19.9**
IR69A × Giza 178 ‡	-0.9ns	-1.7ns	1.3*	3.9*	31.0**	-0.5ns	-4.2ns	-1.7ns	5.9ns	-5.4**	-0.9ns	2.4ns	-5.0ns	-20.2**
L.S.D. 5%	2.2	2.5	1.0	3.0	2.6	0.9	21.0	23.3	0.8	1.0	5.6	0.5	0.1	0.7
L.S.D. 1%	3	3.3	1.3	4	3.5	1.2	28.2	31.4	1.1	1.3	7.5	0.6	0.1	0.9

*: The traits were recorded on maintainer lines which were related to CMS lines

‡: Hybrid check

PF : Pollen fertility percentage, SF: Spikelet fertility percentage, HD: Days to 50% heading (day), PH: Plant height (cm), PL: Panicle length (cm), P/P: Number of panicles per plant, Sp/P: Number of spikelets per panicle, FG/P: Number of filled grains per panicle, PW: Panicle weight (g), 1000-GW: 1000-grain weight (g), GY/P: Grain yield per plant (g), AL: Anther length (mm), AB: Anther breadth (mm), and FL: Filament length (mm),

Table S7. Estimates of the percentage of heterosis over better-parent (BP) for grain quality traits of studied hybrids.

Traits / Crosses	Grain length	Grain width	Grain types	Kernel length	Kernel width	Kernel types	Grain elongation	Hulling (%)	Milling (%)	Head rice (%)
G46A × NRL79	9.8**	3.8ns	4.5ns	2.6ns	2.8ns	-2.3ns	-40.0**	-1.2ns	-2.9*	-10.2**
IR69A × NRL79	8.6**	-4.9*	14.0**	4.9**	-6.9**	12.5**	-21.6**	-1.6ns	-4.2**	-7.4**
G46A × NRL80	-3.0ns	2.7ns	-5.6ns	4.0*	-1.3ns	4.8ns	-25.9**	-0.3ns	0.7ns	2.9ns
IR69A × NRL80	1.7ns	-3.8ns	5.6ns	7.3**	-1.6ns	9.1**	-41.6**	-4.5**	-8.5**	-11.4**
G46A × NRL81	-1.7ns	9.7**	-12.7**	7.6**	6.9**	0.9ns	-57.4**	-3.3**	-0.7ns	0.6ns
IR69A × NRL81	1.9ns	3.3ns	-1.3ns	2.5ns	0.6ns	2.0ns	-42.2**	-3.0**	-0.2ns	-16.9**
G46A × NRL82	-1.7ns	-0.5ns	-1.2ns	7.9**	-5.8**	13.9**	-57.7**	-0.6ns	3.0*	8.4**
IR69A × NRL82	-1.5ns	-4.7*	3.4ns	7.9**	-10.9**	21.2**	-50.3**	-2.3*	-2.3*	-19.9**
G46A × Giza 178	4.1*	2.1ns	-10.1**	8.6**	0.9ns	6.5*	7.9**	-2.0*	-3.4**	-8.2**
IR69A × Giza 178 ‡	6.7**	2.0ns	4.6ns	11.4**	4.2ns	7.2*	-13.8**	-2.8**	-3.3**	-15.3**
L.S.D. 5%	0.3	0.1	0.2	0.2	0.1	0.1	3.7	1.5	1.7	3.1
L.S.D. 1%	0.4	0.2	0.2	0.2	0.2	0.2	5	2.1	2.2	4.1

*: The traits were recorded on maintainer lines which were related to CMS lines ‡: Hybrid check.

Table S8. Estimates of the percentage of heterosis over mid-parents (MP) for grain yield, its contributing and floral traits of studied hybrids.

Traits / Crosses	PF (%)	SF (%)	DH	PH (cm)	P/P	PL (cm)	Sp/P	FG/P	PW (g)	1000 GW (g)	GY/P (g)	AL	AB	FL
G46A × NRL79	-0.4ns	-0.2ns	11.0**	10.1**	32.5**	0.3ns	-2.9ns	-3.3ns	11.7ns	-0.8ns	4.5ns	6.8ns	4.0ns	3.2ns
IR69A × NRL79	-0.4ns	-0.9ns	1.5**	-3.8**	104.7**	2.8ns	-4.8ns	-6.7ns	-6.7ns	-4.1*	21.8**	9.2ns	11.6ns	2.5ns
G46A × NRL80	-2.8**	-3.3**	10.8**	7.0**	117.0**	3.1ns	30.4**	19.4**	-0.8ns	0.9ns	44.4**	5.7ns	6.6ns	4.0ns
IR69A × NRL80	4.1**	4.2**	2.0**	-4.1**	-9.0ns	-7.7**	-25.8**	-22.2**	-26.9**	3.1ns	-2.8ns	9.6ns	9.5ns	3.3ns
G46A × NRL81	2.1*	1.9ns	10.3**	20.5**	97.1**	14.9**	18.5**	19.8**	41.6**	9.7**	66.2**	1.5ns	2.9ns	6.1ns
IR69A × NRL81	0.8ns	-2.0ns	1.6**	-7.4**	-18.7**	8.3**	8.1ns	4.7ns	2.6ns	6.4**	-9.9*	2.8ns	5.9ns	5.6ns
G46A × NRL82	-1.6ns	-2.8*	8.2**	6.7**	71.7**	-4.0*	0.9ns	-8.3ns	-12.7ns	8.0**	-21.9**	6.7ns	8.1ns	7.5ns
IR69A × NRL82	-2.7*	-4.0**	2.4**	-13.4**	22.4**	-9.3**	-1.7ns	-11.8ns	-21.7**	1.2ns	-16.3**	7.7ns	12.8*	7.4ns
G46A × Giza 178	-2.7*	-2.0ns	12.9**	29.6**	27.0**	4.6*	80.3**	77.8**	25.2**	8.0**	21.7**	7.5ns	9.8ns	-1.3ns
IR69A × Giza	-0.7ns	-1.5ns	0.2ns	3.2*	35.1**	1.5ns	5.6ns	8.8ns	7.5ns	3.5ns	5.7ns	4.4ns	-2.6ns	-

178 ‡														2.9ns
L.S.D. 5%	1.93	2.13	0.86	2.60	2.22	0.80	18.18	20.21	0.71	0.84	4.83	0.42	0.05	0.58
L.S.D. 1%	2.59	2.86	1.15	3.49	2.99	1.07	24.44	27.16	0.95	1.13	6.49	0.56	0.07	0.78

*: The traits were recorded on maintainer lines which were related to CMS lines ‡: Hybrid check.

PF : Pollen fertility percentage, SF: Spikelet fertility percentage, HD: Days to 50% heading (day), PH: Plant height (cm), PL: Panicle length (cm), P/P: Number of panicles per plant, Sp/P: Number of spikelets per panicle, FG/P: Number of filled grains per panicle, PW: Panicle weight (g), 1000-GW: 1000-grain weight (g), GY/P: Grain yield per plant (g), AL: Anther length (mm), AB: Anther breadth (mm), and FL: Filament length (mm),

Table S9. Estimates of the percentage of heterosis over mid-parents (MP) for grain quality traits of studied hybrids.

Traits / Crosses	Grain length	Grain width	Grain types	Kernel length	Kernel width	Kernel types	Grain elongation	Hulling (%)	Milling (%)	Head rice (%)
G46A × NRL79	-0.5ns	4.3*	-4.7ns	1.6ns	4.4*	-2.8ns	-35.7**	-0.2ns	-0.1ns	-9.5**
IR69A × NRL79	-0.1ns	2.1ns	-3.3ns	0.3ns	2.7ns	-3.9ns	-7.6**	-1.2ns	-2.8**	-5.0*
G46A × NRL80	-7.2**	3.0ns	-9.9**	3.7*	1.6ns	2.1ns	-20.0**	2.0*	4.2**	5.1*
IR69A × NRL80	-1.3ns	4.1*	-6.0*	1.3ns	4.3*	-3.6ns	-30.7**	-2.9**	-6.6**	-6.6**
G46A × NRL81	-5.3**	11.2**	-14.7**	4.8**	7.5**	-2.4ns	-49.2**	-0.5ns	4.0**	3.4ns

IR69A × NRL81	-0.4ns	10.1**	-10.0**	-0.3ns	8.9**	-9.1**	-25.1**	-0.8ns	3.0**	-11.9**
G46A × NRL82	-4.9**	1.6ns	-6.5*	7.6**	-2.7ns	10.5**	-50.4**	1.4ns	6.2**	10.3**
IR69A × NRL82	-3.3*	4.8**	-8.8**	2.5ns	-0.1ns	0.5ns	-36.5**	-1.0ns	-0.7ns	-15.8**
G46A × Giza 178	-3.0ns	8.5**	-10.6**	8.0**	6.5**	1.1ns	10.5**	0.1ns	-0.2ns	-5.5**
IR69A × Giza 178 ‡	0.8ns	3.6ns	-2.8ns	4.9**	7.6**	-2.5ns	-6.5**	-1.2ns	-1.5ns	-9.9**
L.S.D. 5%	0.24	0.10	0.13	0.15	0.10	0.10	3.20	1.33	1.44	2.66
L.S.D. 1%	0.33	0.14	0.18	0.2	0.14	0.14	4.3	1.79	1.94	3.58

*: The traits were recorded on maintainer lines which were related to CMS lines ‡: Hybrid check.

Table S10. Estimates of the percentage of standard heterosis (SH) for grain yield, its contributing and floral traits of studied hybrids.

Traits / Crosses	PF (%)	SF (%)	DH	PH (cm)	P/P	PL (cm)	Sp/P	FG/P	PW (g)	1000 GW (g)	GY/P (g)	AL	AB	FL
G46A × NRL79	0.9ns	1.6ns	0.01ns	2.9*	-12.3ns	7.6**	40.6**	33.1**	37.2**	5.7**	24.6**	24.9ns	13.2ns	41.3**
IR69A × NRL79	0.9ns	0.9ns	1.4**	-3.1*	54.8**	7.6**	25.1**	16.3*	13.2ns	0.7ns	47.2**	28.6*	26.3**	38.2**
G46A × NRL80	-1.6ns	-1.3ns	1.0*	3.6*	50.2**	11.2**	70.9**	50.2**	23.4*	6.7**	66.9**	7.3ns	13.2ns	40.7**

IR69A × NRL80	5.40**	6.4**	2.9**	-0.1ns	-28.5**	-3.0ns	-12.6ns	-12.3ns	-10.0ns	7.4**	13.8ns	12.1ns	21.1**	37.6**
G46A × NRL81	5.2**	6.2**	1.0*	16.8**	59.3**	12.6**	30.0**	29.3**	49.5**	18.2**	125.1**	-0.7ns	10.5ns	41.7**
IR69A × NRL81	3.9**	2.2ns	3.0**	-3.4*	-26.7**	3.2ns	4.1ns	-0.6ns	6.9ns	13.0**	23.4**	1.4ns	18.4*	38.8**
G46A × NRL82	-0.7ns	-1.0ns	0.003ns	11.9**	44.8**	7.6**	21.6**	6.5ns	0.4ns	20.8**	7.3ns	34.5**	28.9**	52.6**
IR69A × NRL82	-1.7ns	-2.3ns	4.6**	-2.8ns	14.8*	-0.9ns	5.3ns	-9.0ns	-11.0ns	11.6**	16.2*	36.7**	39.5**	50.2**
G46A × Giza 178	-2.0ns	-0.6ns	1.7**	16.1**	-18.0*	5.9**	95.0**	86.7**	18.1ns	6.0**	13.2ns	2.1ns	7.9ns	3.7ns
IR69A × Giza 178 ‡	0.0ns	0.00ns	0.0ns	0.0ns	0.0ns	0.0ns	0.0ns	0.0ns	0.0ns	0.0ns	0.0ns	0.0ns	0.0ns	0.0ns
L.S.D 5%	2.2	2.5	1	3.0	2.6	0.9	21.0	23.3	0.8	1.0	5.6	0.5	0.1	0.7
L.S.D 1%	3	3.3	1.3	4	3.5	1.2	28.2	31.4	1.1	1.3	7.5	0.6	0.1	0.9

*: The traits were recorded on maintainer lines which were related to CMS lines ‡: Hybrid check.

PF: Pollen fertility percentage, SF: Spikelet fertility percentage, HD: Days to 50% heading (day), PH: Plant height (cm), PL: Panicle length (cm), P/P: number of panicles per plant, Sp/P: Number of spikelets per panicle, FG/P: Number of filled grains per panicle, PW: Panicle weight (g), 1000-GW: 1000-grain weight (g), GY/P: Grain yield per plant (g), AL: Anther length (mm), AB: Anther breadth (mm), and FL: Filament length (mm),

Table S11. Estimates of the percentage of standard heterosis (SH) for grain quality traits of studied hybrids.

Traits	Grain	Grain	Grain	Kernel	Kernel	Kernel	Grain	Hulling (%)	Milling (%)	Head rice (%)

Crosses	length	width	types	length	width	types	elongation			
G46A × NRL79	-2.2ns	15.5**	-15.3**	-6.9**	13.8**	-18.5**	-15.6**	-0.8ns	-0.3ns	-0.2ns
IR69A × NRL79	-3.3ns	4.7*	-7.6**	-3.0ns	3.1ns	-6.1*	10.2**	-1.2ns	-1.6ns	1.4ns
G46A × NRL80	-4.4*	14.9**	-16.7**	-6.3**	6.0*	-11.8**	5.9*	2.7**	4.6**	19.3**
IR69A × NRL80	0.3ns	7.7**	-6.7*	-3.3*	-0.3ns	-3.3ns	-16.6**	-1.6ns	-4.9**	2.7ns
G46A × NRL81	-1.8ns	22.1**	-19.4**	-2.4ns	14.8**	-15.0**	-23.3**	0.8ns	5.7**	18.1**
IR69A × NRL81	1.8ns	11.9**	-8.9**	-2.1ns	6.7**	-8.3**	4.0ns	1.1ns	6.2**	-2.5ns
G46A × NRL82	-0.9ns	15.5**	-14.2**	-2.1ns	8.1**	-9.7**	-26.9**	1.8ns	6.2**	24.9**
IR69A × NRL82	-0.7ns	10.7**	-10.2**	-1.5ns	2.3ns	-3.9ns	-14.0**	0.1ns	0.8ns	-7.7**
G46A × Giza 178	-2.4ns	13.6**	-14.1**	-2.5ns	8.3**	-10.3**	31.4**	0.7ns	-0.1ns	8.3**
IR69A × Giza 178 ‡	0.0ns	0.0ns	0.0ns	0.0ns	0.0ns	0.0ns	0.0ns	0.0ns	0.0ns	0.0ns
L.S.D 5%	0.3	0.1	0.2	0.2	0.1	0.1	3.7	1.5	1.7	3.1
L.S.D 1%	0.4	0.2	0.2	0.2	0.2	0.2	5	2.1	2.2	4.1

*: The traits were recorded on maintainer lines which were related to CMS lines ‡: Hybrid check.
